# Impact of improved stroke green channel process on the delay of intravenous thrombolysis in patients with acute cerebral infarction during the COVID-19 pandemic: An observational study

**DOI:** 10.3389/fneur.2022.998134

**Published:** 2022-09-26

**Authors:** Qiwei Wang, Yan Wang, Yongpeng Wang, Qianqian Bi, Quanbin Zhang, Feng Wang

**Affiliations:** ^1^Departments of Neurology, Seventh People's Hospital of Shanghai University of Traditional Chinese Medicine, Shanghai, China; ^2^Department of Neurology, Shanghai General Hospital of Shanghai Jiao Tong University School of Medicine, Shanghai, China; ^3^Department of Neurosurgery, Shanghai Tenth People's Hospital, Tongji University School of Medicine, Shanghai, China

**Keywords:** acute cerebral infarction, COVID-19, improved green channel for intravenous thrombolysis, intravenous thrombolysis, time delay

## Abstract

**Objective:**

This study analyzed the impact of the improved stroke green channel process on the delay of intravenous thrombolysis in patients with acute cerebral infarction under coronavirus disease 2019 (COVID-19) prevention and control measures.

**Methods:**

We included 57 patients from the stroke center of the Seventh People's Hospital of Shanghai University of Traditional Chinese Medicine before the improvement of the stroke green channel process (March–July 2019), as well as 94 patients during the severe acute respiratory syndrome coronavirus 2 (SARS-CoV-2) outbreak (March–July 2020) and 68 patients during the Omicron variant outbreak (March–July 2022) after the improvement of stroke green channel process. The door-to-needle time (DNT), door-to-imaging time (DIT), and door-to-test completion time were compared among the three groups. We analyzed the impact of this process improvement in the emergency green channel during the pandemic on the delay of intravenous thrombolysis.

**Results:**

This study included a total of 229 patients with acute cerebral infarction who went through the green channel for intravenous thrombolysis (57 in the pre-pandemic group, 94 in the SARS-CoV-2 outbreak group, and 68 in the Omicron outbreak group). The percentages of patients undergoing intravenous thrombolysis in the pre-pandemic, SARS-CoV-2 outbreak, and Omicron outbreak groups differed significantly (19.32%, 22.27%, and 28.94%, respectively, *P* = 0.029). Compared to the pre-pandemic group, the National Institutes of Health Stroke Scale (NIHSS) score at admission was significantly higher in the Omicron outbreak group (7.71 ± 7.36 in the Omicron outbreak group vs. 5.00 ± 4.52 in the pre-pandemic group) (*P* = 0.026) but not in the SARS-CoV-2 outbreak group (4.79 ± 5.94 in the SARS-CoV-2 outbreak group vs. 5.00 ± 4.52 in the pre-pandemic group, *P* = 0.970). Significantly higher proportions of patients undergoing emergency intravenous thrombolysis came to the hospital by ambulance in the SARS-CoV-2 and Omicron outbreak groups compared to the pre-pandemic group (38.6% in the pre-pandemic group, 51.1% in the SARS-CoV-2 outbreak group, and 82.4% in the Omicron outbreak group, *P* < 0.001). Compared to the pre-pandemic group, the DIT was significantly higher in the SARS-CoV-2 outbreak group (22.42 ± 7.62 min in the SARS-CoV-2 outbreak group vs. 18.91 ± 8.23 min in the pre-pandemic group, *P* =0.031) but not the Omicron outbreak group (20.35 ± 10.38 min in the Omicron outbreak group vs. 18.91 ± 8.23 min in the pre-pandemic group, *P* = 0.543). The door-to-test completion time was significantly longer in the SARS-CoV-2 and Omicron outbreak groups compared to that in the pre-pandemic group (78.37 ± 25.17 min in the SARS-CoV-2 outbreak group, 92.60 ± 25.82 min in the Omicron outbreak group vs. 65.11 ± 22.35 min in the pre-pandemic group, P < 0.001); however, the DNT in the SARS-CoV-2 and Omicron outbreak groups did not differ significantly from those in the pre-pandemic group (both P > 0.05).

**Conclusion:**

During the two periods of the COVID-19 outbreak (SARS-CoV-2 and Omicron), after the improvement of the green channel for intravenous thrombolysis, there might be some delay in in-hospital DIT during the SARS-CoV-2 outbreak, however, the in-hospital delay indicator DNT for intravenous thrombolysis were not affected.

## Introduction

Acute cerebral infarction is characterized by high incidence, disability, and mortality rates. Patients are often left with motor-sensory, language function, and cognitive impairments, which not only significantly reduce their quality of life but also impose great burdens on families and society. Currently, intravenous thrombolytic therapy is the most effective treatment for early-stage cerebral infarction. This therapy recanalizes the occluded blood vessels and restores cerebral blood flow, helping to save damaged neurons in the brain, thus minimizing the degree of patient disability. However, the administration of intravenous thrombolytic therapy has a strict time window of 4.5 h after disease onset, which can be extended to 9 h for patients who pass a strict screening process (1). Thrombolysis beyond this time window not only fails to improve neurological function but also may induce serious complications such as reperfusion injury. The prognosis of patients with acute cerebral infarction is closely related to the timing of thrombolysis (2, 3). The construction of the stroke green channel aims to shorten the time delay for the administration of intravenous thrombolysis and to achieve a good patient prognosis.

In December 2019, a pneumonia outbreak caused by a novel coronavirus occurred in Wuhan City, Hubei Province, China, and surrounding areas. The World Health Organization (WHO) announced the name of the novel coronavirus pneumonia, coronavirus disease 2019 (COVID-19), on February 11, 2020. At the same time, the International Committee on Taxonomy of Viruses declared that the novel coronavirus was named severe acute respiratory syndrome coronavirus 2 (SARS-CoV-2). The COVID-19 outbreak spread rapidly across the country, and Shanghai launched a Level 1 response to major public health emergencies to control the spread of the virus. After the WHO announced on November 25, 2021, that the Omicron (B.1.1.529) variant of SARS-CoV-2 was detected for the first time in South Africa, this variant has led to a global surge of newly diagnosed cases of COVID-19. An outbreak of the Omicron variant occurred in Shanghai in March 2022. To prevent viral spread within healthcare facilities and ensure that patients with acute stroke were treated effectively and in promptly, new requirements were introduced for stroke green channel management (4). Considering the need for pandemic prevention and control, our hospital has improved and optimized the emergency stroke green channel accordingly since March 2020. Further improvement was implemented in March 2022 to address the Omicron variant. The present study explored whether the improvement and optimization of the stroke green channel process under the prevention and control of the COVID-19 pandemic would affect the timing for intravenous thrombolysis in patients with acute cerebral infarction.

## Materials and methods

### Subjects

The data of 219 patients with acute ischemic stroke who were sequentially admitted to the stroke center of the Seventh People's Hospital of Shanghai University of Traditional Chinese Medicine through the stroke green channel process from March 1 to July 31, 2019 (pre-pandemic group), March 1 to July 31, 2020 (SARS-CoV-2 outbreak group), and March 1 to July 31, 2022 (Omicron outbreak group) were included in this study.

The SARS-CoV-2 outbreak group adopted the initial improved stroke green channel process, while the Omicron outbreak group adopted the advanced improved stroke green channel process. The pre-hospital and in-hospital delays of the patients in these two groups were compared to those of patients in the pre-pandemic group.

## Methods

### Inclusion criteria

The inclusion criteria were as follows: acute ischemic stroke diagnosed by head computed tomography (CT); within 4.5 h of symptom onset; age ≥18 years; informed consent signed by family members; patients who met the diagnostic criteria of the “Chinese Guidelines for Diagnosis and Treatment of Acute Ischemic Stroke 2018; and underwent intravenous thrombolysis. The exclusion criteria were patients with acute cerebral infarction who did not undergo intravenous thrombolysis.

### Emergency stroke green channel process

#### Before green channel improvement

The stroke center was established in 2015. Before the COVID-19 outbreak, no epidemic prevention measures had been adopted for the emergency stroke green channel. Upon patient arrival at the emergency room, the nurse at the reception desk triaged the patient according to the symptoms, and patients with suspected stroke were directed into the emergency room. The emergency physician in the stroke center recorded the patient medical history and conducted physical examinations and then prescribed assessments, including head CT scan, hematology tests, electrocardiogram (ECG), etc. Subsequently, the emergency physician analyzed the examination results and confirmed the diagnosis of acute ischemic stroke. In patients who did not exceed the time window for intravenous thrombolysis, the current condition was explained in detail to the patient's family members, and informed consent for intravenous thrombolysis was requested. The patients received thrombolytic therapy in the emergency room under ECG monitoring and were then admitted to the stroke unit for further diagnosis and treatment.

#### After green channel improvement

**Figure 1:**
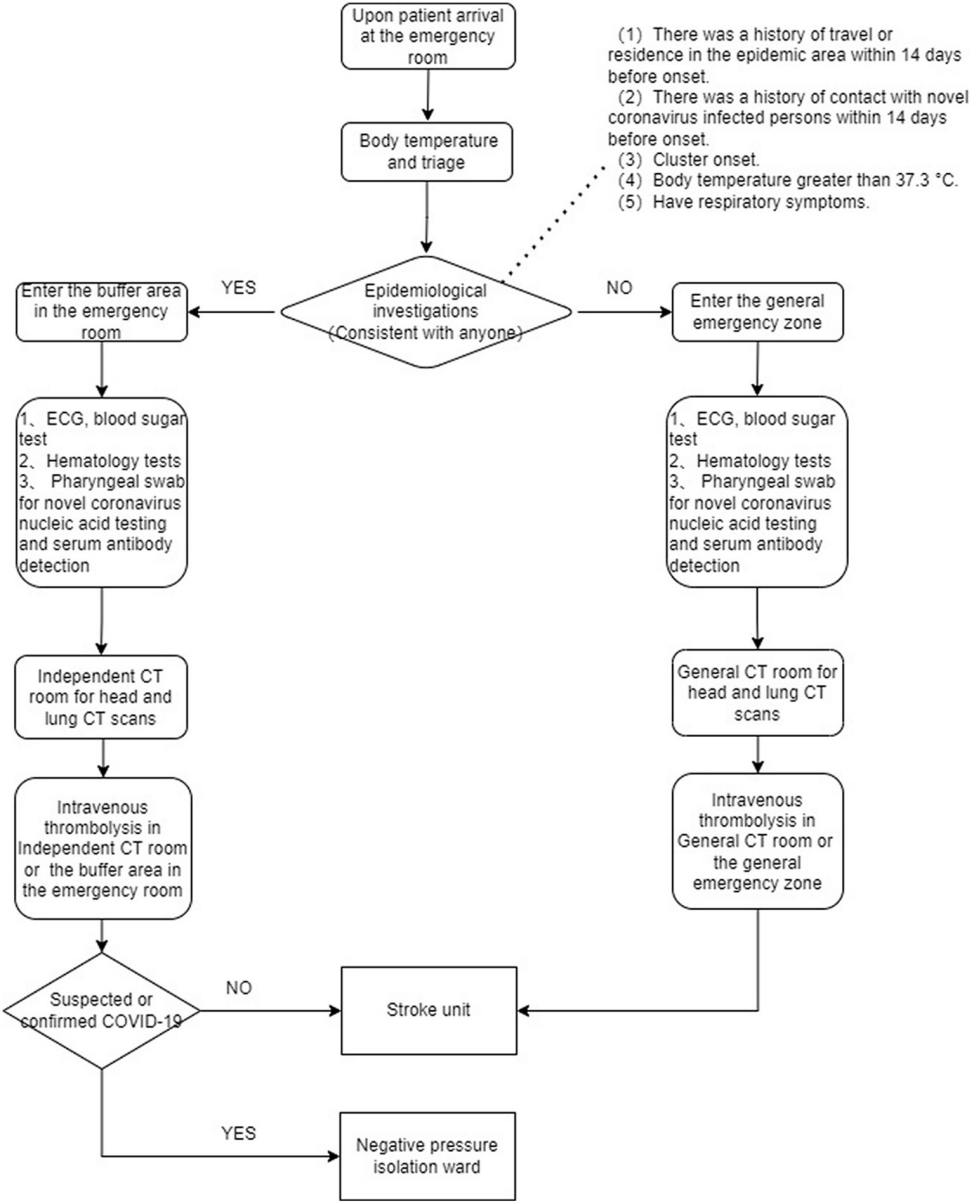
The improved process of the emergency stroke green channel after the pandemic onset.

COVID-19 first emerged in China in November 2019 and gradually spread across the entire country. On February 20, 2020, we improved the green channel process to prevent the spread of the virus and to minimize the impact on in-hospital delays. The improved green channel process was adopted at the hospital from February 20, 2020 to February 28, 2022. Upon patient arrival at the emergency room, additional full-time nurses performed epidemiological investigations, body temperature measurements, and triage. Patients with suspected stroke with no travel history to high-risk epidemic areas and no fever followed the previous stroke green channel process, with the addition of lung CT scan, novel coronavirus nucleic acid testing *via* pharyngeal swab, and serum antibody detection. Patients with a travel history to epidemic areas or fever were first admitted to the buffer area in the emergency room. The buffer area was relatively isolated, with independent nurses and the medical staff wearing level two protection to enter the buffer area (work clothes, work cap, medical protective mask, goggles or face shield, and disposable isolation gown). The emergency physician in the stroke center recorded the patient's medical history and performed physical examinations in the buffer area, while ECG, blood pressure, pulse, respiration, and blood oxygen saturation (SPO_2_) were monitored. ECG, blood sugar test, hematology tests, pharyngeal swab for novel coronavirus nucleic acid testing and serum antibody detection were performed at the bedside. The buffer area could directly access an independent CT room for head and lung CT scans. Patients eligible for thrombolysis after examination were returned to the buffer area. The emergency physician in the stroke center talked with the patient and family members and requested informed consent for thrombolysis. The patient then received intravenous thrombolysis and was observed. Patients diagnosed with or with suspected COVID-19 based on follow-up findings were admitted to the negative pressure isolation unit after intravenous thrombolysis. Patients in whom COVID-19 was excluded were admitted to the stroke unit for further treatment (Figure 1).

#### Advanced improvement of the green channel

The Omicron variant outbreak occurred in Shanghai in March 2022. To address the issues of faster transmission and higher vaccine resistance associated with the Omicron variant, further improvement was made to the green channel on March 1, 2022. Based on the original pandemic prevention measures, the COVID-19 antigen detection was added upon patient admission to the hospital. Four physicians from the stroke center were stationed in the emergency room, all of whom were required to see patients while wearing level two protection in shifts every 8 h; the other procedures were consistent with the improved green channel process in 2.2.2.

### Observation indicators

In this study, the door-to-needle time (DNT), door-to-imaging time (DIT), and door-to-test completion time were used as the main time indicators of in-hospital delay of the stroke green channel. The Modified Rankin Scale (mRS) score at 30 days, incidence of spontaneous intracerebral hemorrhage (sCIH) and 30-day mortality were adopted as indicators for the observation of patient outcomes. Indicator variables subjected to statistical analysis in this study included demographic data (age and sex); presence or absence of history of hypertension, diabetes, coronary heart disease, atrial fibrillation, and stroke; mode of hospital arrival (*via* the emergency medical service or other methods); and stroke severity assessed using the National Institutes of Health Stroke Scale (NIHSS). The main time indicators were defined according to international standards as follows: (1) onset time: time of onset of the current stroke symptoms of the patient (for patients with an unclear onset time, the last-known normal time was used); (2) door time: time at which the patient arrived at the emergency room for registration; (3) head imaging time: time at which the plain CT scan of the head had been completed and the images had been obtained; (4) test completion time: time at which the laboratory provided all test reports; and (5) intravenous thrombolysis time: time of initiation of intravenous thrombolytic therapy.

### Date collection and patient follow

During the hospital stay of each patient, the following data were recorded by a neurologist: patient age, sex, past histories, mode of transportation to the hospital, NIHSS score at admission, NIHSS score at discharge, DNT, DIT, door-to-test completion time, and whether sCIH had occurred. For each patient or patient's surrogate, reliable contact information was obtained to assess outcome in person (eg., clinic follow-up) or by telephone between 30 days from the incident stroke. The mRS score was obtained at 30 days (+7 days allowed to establish contact). Patients who were lost to follow-up received the last recorded mRS or an mRS derived from the last documented neurological evaluation as their final score.

### Statistical analysis

Statistical analysis was performed using IBM SPSS Statistics for Windows, version 25.0. The quantitative data were expressed as $\bar{\text{x}}$ ± s, and χ ^2^ tests were used for comparisons among the three groups. Qualitative data were expressed as frequencies, and comparisons among the three groups were performed using Fisher's exact tests. Pairwise comparisons among the three groups were performed using Dunnett's tests. *P* < 0.05 indicated statistical significance.

### Ethics statement

The studies involving human participants were reviewed and approved by Seventh People's Hospital of Shanghai University of Traditional Chinese Medicine. The patients/participants provided their written informed consent to participate in this study.

## Results

### Basic clinical data of the three patient groups

A total of 295 patients with acute cerebral infarction were admitted to our hospital between March and July 2019, before the pandemic onset. Of these, 57 patients (19.32%) underwent intravenous thrombolysis in the emergency room. A total of 422 patients with acute cerebral infarction were admitted between March and July, during the SARS-CoV-2 outbreak. Of these, 94 patients 22.27%) underwent intravenous thrombolysis in the emergency room. A total of 235 patients with acute cerebral infarction were admitted between March and July, during the Omicron outbreak. Of these, 68 patients (28.94%) underwent intravenous thrombolysis in the emergency room (*P* = 0.029).

The baseline data of the three groups of patients are shown in Table 1. Patient age; sex; and histories of hypertension, diabetes, coronary heart disease, atrial fibrillation, and stroke did not differ significantly among the groups (all *P* > 0.05). The NIHSS scores at admission did not differ significantly between the SARS-CoV-2 outbreak group and the pre-pandemic group, but was significantly higher in the Omicron outbreak group than that in the pre-pandemic group (7.71 ± 7.36 vs. 5.00 ± 4.52, *P* = 0.026). The NIHSS scores did not differ significantly among the three patient groups at discharge (pre-pandemic group 3.84 ± 4.57, SARS-CoV-2 outbreak group 4.73 ± 7.20, Omicron outbreak group 5.22 ± 7.07, *P* = 0.501). The proportions of patients undergoing emergency intravenous thrombolysis who came to the hospital by ambulance were significantly higher during the COVID-19 pandemic (pre-pandemic group 38.6%, SARS-CoV-2 outbreak group 51.1%, Omicron outbreak group 82.4%, *P* < 0.001).

**Table 1:** Baseline information of three patients groups undergoing intravenous thrombolysis.

	**Pre-pandemic group** ***N* = 57**	**SARS-CoV-2 outbreak group** ***N* = 94**	**Omicron outbreak group** ***N* = 68**	**P value**
Age (years)	65.84 ± 14.35	66.98 ± 14.21	66.41 ± 13.70	0.889
Male sex	36 (63.2%)	52 (55.3%)	49 (72.1%)	0.094
History of hypertension	39 (68.4%)	56 (59.6%)	51 (75.0%)	0.115
History of diabetes	13 (22.8%)	20 (21.3%)	15 (22.1%)	0.975
History of coronary heart disease	10 (17.5%)	18 (19.1%)	15 (22.1%)	0.808
History of atrial fibrillation	2 (3.5%)	10 (10.6%)	8 (11.8%)	0.223
History of cerebral infarction	12 (21.1%)	13 (13.8%)	13 (19.1%)	0.471
NIHSS score at admission	5.00 ± 4.52	4.79 ± 5.94	7.71 ± 7.36	0.007
NIHSS score at discharge	3.84 ± 4.57	4.73 ± 7.20	5.22 ± 7.07	0.501
Taking an ambulance to the hospital	22 (38.6%)	48 (51.1%)	56 (82.4%)	< 0.001

NIHSS, the National Institute of Health Scale Score.

### Comparison of the delay indicators for intravenous thrombolysis in the three patient groups

The DNT did not differ significantly among the three patient groups (39.96 ± 10.75 min in the pre-pandemic group, 46.07 ± 22.83 min in the SARS-CoV-2 outbreak group, and 39.92 ± 17.12 min in the Omicron outbreak group, *P* = 0.064).

The DIT in the SARS-CoV-2 outbreak group was significantly longer than that in the pre-pandemic group (22.42 ± 7.62 vs. 18.91 ± 8.23 min, *P* = 0.031), but did not differ significantly between the Omicron outbreak group and pre-pandemic group (20.35± 10.38 min vs. 18.91 ± 8.23 min, *P* = 0.543).

The door-to-test completion time in the Omicron outbreak group was significantly longer than that in the pre-pandemic group (92.60 ± 25.82 min vs. 65.11 ± 22.35 min, *P* < 0.001) and also visible between the SARS-CoV-2 outbreak group and the pre-pandemic group (78.37 ± 25.17 min vs. 65.11 ± 22.35 min, *P* = 0.003) (Tables 2, 3).

**Table 2:** Comparisons of in-hospital delay indicators in the three groups of patients undergoing intravenous thrombolysis.

	**Pre-pandemic group *N =* 57**	**SARS-CoV-2 outbreak group *N =* 94**	**Omicron outbreak group *N =* 68**	**P value**
DIT (min)	18.91 ± 8.23	22.42 ± 7.62	20.35 ± 10.38	0.049
DNT (min)	39.96 ± 10.75	46.07 ± 22.83	39.92 ± 17.12	0.064
DNT < 60 min	54 (94.7%)	80 (85.1%)	62 (91.2%)	0.150
Door-to-test completion time (min)	65.11 ± 22.35	78.37 ± 25.17	92.60 ± 25.82	< 0.001

NIHSS, the National Institute of Health Scale Score; DNT, door-to-needle time; DIT, door-to-imaging time.

**Table 3:** Further comparisons by Dunnett's test.

	**Group**	**Group**	***P*-value**
NIHSS score at admission	SARS-CoV-2 outbreak group	Pre-pandemic group	0.970
	Omicron outbreak group	Pre-pandemic group	0.026
DIT	SARS-CoV-2 outbreak group	Pre-pandemic group	0.031
	Omicron outbreak group	Pre-pandemic group	0.543
Door-to-test completion time	SARS-CoV-2 outbreak group	Pre-pandemic group	0.003
	Omicron outbreak group	Pre-pandemic group	< 0.001

NIHSS, the National Institute of Health Scale Score; DIT, door-to-imaging time.

### Comparison of outcome indicators of the three groups

At the 1-month follow-up, the proportion of patients with mRS scores of 0–2 did not differ significantly among the three groups (82.5% in the pre-pandemic group, 76.6% in the SARS-CoV-2 outbreak group, and 73.5% in the Omicron outbreak group, *P* = 0.488). The incidence of sICH was not significantly different among the three groups (1.8% in the pre-pandemic group, 2.7% in the SARS-CoV-2 outbreak group, and 3.2% in the Omicron outbreak group, *P* = 0.151). Significant differences in 30-day mortality were also absent among the three groups (1.8% in the pre-pandemic group, 2.1% in the SARS-CoV-2 outbreak group, and 2.9% in the Omicron outbreak group, *P* = 0.899) (Table 4).

**Table 4:** Comparisons of outcome indicators in the three groups of patients undergoing intravenous thrombolysis.

	**Pre-pandemic group *N =* 57**	**SARS-CoV-2 outbreak group *N =* 94**	**Omicron outbreak group *N =* 68**	***P*-value**
				
MRS 0-2	47 (82.5%)	72 (76.6%)	50 (73.5%)	0.488
sICH	1 (1.8%)	6 (2.7%)	7 (3.2%)	0.151
30 days mortality	1 (1.8%)	2 (2.1%)	2 (2.9%)	0.899

MRS, Modified Rankin Scale; sICH,symptomatic Intracranial Hemorrhage.

## Discussion

The COVID-19 pandemic is the largest public health crisis in a century, with an estimated 541 million infections and 6.33 million deaths as of June 28, 2022 (5). The novel coronavirus pneumonia caused by SARS-CoV-2 infection appeared in December 2019, with its widespread dissemination of SARS-CoV-2 and the large number of infected patients attracting global attention. At present, SARS-CoV-2 has evolved into multiple variants, including α (B.1.1.7), Beta (B.1.351), Gamma (P.1), Delta (B.1.617.2), and the currently dominant variant Omicron (B.1.1.529). Due to the global spread of the Omicron variant, the cumulative number of infections in Shanghai from March to July 2022 exceeded 600,000. Compared with the other variants, the Omicron variant is the most highly mutated variant, spreads faster than the original virus strain and other VOCs, and poses a higher risk of reinfection than the other variants (6, 7), thus posing a greater challenge for outbreak prevention and control.

Emergency medical service resources were heavily utilized during the COVID-19 pandemic, with a 330% increase in the number of calls to the emergency medical system in some places (8). The results of the present study showed an increased proportion of patients with acute cerebral infarction who visited the clinic by ambulance during the SARS-CoV-2 outbreak. It was also observed that the proportion of patients with acute cerebral infarction who arrived at the hospital *via* emergency medical services increased further during the Omicron outbreak, which may be due to the strict traffic control measures and movement restrictions imposed in Shanghai during this period.

The COVID-19 pandemic has an inevitable impact on the management of stroke patients. Some studies have shown a decreased rate of stroke patients intravenous thrombolysis during the pandemic, which might be related to the delayed access or even reluctance of stroke patients to seek medical care during this time (9). However, some studies also showed that the reperfusion therapy rate was not greatly affected during the pandemic (10). Our study indicates that the number of patients with acute cerebral infarction and proportion of acute cerebral infarction patients who received intravenous thrombolytic therapy were increased during the SARS-CoV-2 outbreak compared with the pre-pandemic period. Previous studies have shown that first-line neurologist decision, Laboratory and neuroimaging in nearest Location can increase the rate of intravenous thrombolysis (11). In our process improvement, we set up a separate CT room and access, and arranged a separate neurologist in the emergency department, which may have played a role. During the Omicron outbreak, the number of patients with acute cerebral infarction decreased significantly compared with the previous period, although the number of patients who underwent intravenous thrombolysis did not show a corresponding decrease, resulting in an increase in the proportion of intravenous thrombolysis. A possible explanation for this phenomenon is that the number of COVID-19 cases during the SARS-CoV-2 outbreak period was relatively low, which did not affect the healthcare seeking behavior of patients. In addition, health education related to stroke has been ongoing; previous research has shown that public health education promotes an increase in the intravenous thrombolysis rate (12). However, the number of COVID-19 cases in Shanghai showed a huge surge during the Omicron outbreak period, prompting the adoption of traffic control measures and movement restrictions by the Shanghai government. Consequently, some patients, especially those with mild symptoms and slow progression who were less likely to experience obstruction of large blood vessels, avoided seeking medical care due to fear of being infected. This resulted in a decrease in the number of acute cerebral infarction patients who sought medical attention, although those with a more severe condition, who were in genuine need of emergency intravenous thrombolysis, were still transported to the hospital *via* emergency medical services.

SARS-CoV-2 is transmitted primarily through respiratory and contact transmission. The safe distance between people is inevitably reduced in a medical setting, which increases the risk of infection. During the COVID-19 pandemic, the optimization of the stroke green channel played a great role in protecting the health environment of medical institutions and the safety of relevant medical personnel, preventing the spread of SARS-CoV-2 in medical institutions, and ensuring that patients with acute stroke received timely and effective treatment. During the SARS-CoV-2 outbreak, we: (1) arranged for trained full-time nurses in the stroke green channel to conduct epidemiological investigations, body temperature measurements, and screening of stroke patients to screen for patients at risk of COVID-19 infection while performing triage; (2) required all medical staff working in the stroke green channel to implement level 2 or higher protection in the emergency room (wearing work clothes, a disposable cap, a medical protective mask [N95 or above], goggles or face shield, a medical protective gown, disposable gloves, and disposable shoe covers) and also required patients and their family members to wear a mask throughout the whole process; (3) set up an independent buffer area in the emergency room to isolate suspected infected patients from non-infected patients and reduce transmission; (4) set up an independent CT room near the emergency buffer area equipped with radiologists. Lung CT scans were performed in patients with suspected infection, who reached the independent CT room through an independent channel; (5) performed pharyngeal swabs for novel coronavirus nucleic acid testing and serum antibody detection. During the Omicron outbreak in 2022, we further optimized the stroke green channel given the highly infectious and insidious characteristics of this variant: (1) Novel coronavirus antigen detection was performed additionally in patients at admission to provide rapid results; (2) a biometric door reader with body temperature detection was set up to facilitate the detection of febrile patients at admission; (3) several doctors stationed in the stroke green channel in shifts were relatively isolated from other areas of the hospital to reduce the risk of transmission. These additional protocols were designed to prevent the spread of the virus inside the hospital and they worked accordingly. During the pandemic, no patients with acute cerebral infarction in the stroke green channel were cross-infected in our hospital. Studies have shown that patients presenting to the hospital with acute neurological symptoms during the COVID-19 pandemic may experience delayed treatment due to in-hospital delays (13, 14). In this study, while the DIT time during the SARS-CoV-2 outbreak increased, the DIT time during the Omicron outbreak did not differ significantly from that before the pandemic, which might be related to the additional COVID-19 screening measures at the beginning of the pandemic. However, with the continuous improvement of the stroke green channel after the normalized prevention and control of the pandemic, and the continuous familiarity of nurses, doctors from the stroke center, and radiologists with the improved process, the impact continued to decrease over time. Moreover, the DNT for intravenous thrombolysis and proportion of DNT < 60 min in the new process for the stroke green channel was not affected by the pandemic, during either the SARS-CoV-2 or Omicron outbreaks This result indicates that the optimization of the green channel process to reduce in-hospital virus transmission did not affect the in-hospital delay of intravenous thrombolysis.

In this study, the door-to-test completion time was prolonged in patients with acute cerebral infarction during the SARS-CoV-2 outbreak and the Omicron outbreak compared to that before the pandemic, a further increase occurred during the Omicron outbreak compared with the SARS-CoV-2 outbreak, which may be due to the increase in antigen and antibody testing for outbreak prevention and control during the SARS-CoV-2 outbreak period. This might be related to the demand for medical resources during the Omicron outbreak and the participation of laboratory physicians in nucleic acid screening in the community, resulting in a decreased number of laboratory physicians in the hospital. Although the door-to-test completion time did not affect the in-hospital delay in intravenous thrombolysis, reduced platelet count and abnormal coagulation function are contraindications to intravenous thrombolysis in some patients (15). Thus, it was necessary to wait for the laboratory test results before starting intravenous thrombolysis, which may have affected the thrombolysis timing.

Our results showed that the incidence of sICH after intravenous thrombolysis and 30-day mortality did not differ significantly among the three groups of patients and were not affected by adjustments made to the green channel process during the pandemic period. To investigate the short-term outcomes of the patients, we performed a 30-day follow-up of the mRS scores of patients. It was found that the good outcome rate (proportion of patients with mRS scores of 0–2) was also not significantly different among the three groups.

This study has certain limitations. First, this was a single-center study with a relatively small sample size, which may not comprehensively reflect the effects of the COVID-19 pandemic on stroke green channels in high-risk areas. However, the hospital had experienced the SARS-CoV-2 and Omicron outbreak stages of the COVID-19 pandemic and implemented corresponding pandemic prevention and control measures according to the different virus transmission characteristics of the two stages. Therefore, our results possess certain reference value, and it is not limited to the COVID-19 virus. Second, there are certain inherent limitations due to the retrospective nature of the study, any observed changes may have been subject to general trends or interventions targeting public health or individual behavior changing. We hope that future prospective studies will be conducted to understand the role of green channel adjustment in epidemic prevention and control. Last, since arterial thrombectomy was not performed in our hospital before 2020, there is a lack of impact of improved green access on in-hospital time delays for arterial thrombectomy during the pandemic.

In conclusion, the results of this study showed that during the two outbreaks of the COVID-19 pandemic (SARS-CoV-2 and Omicron), improvement of the green channel for intravenous thrombolysis might have caused some delay in the in-hospital DIT during the SARS-CoV-2 outbreak, however, it did not affect the in-hospital delay indicator DNT for intravenous thrombolysis during the pandemic.

## Data availability statement

The original contributions presented in the study are included in the article/Supplementary material, further inquiries can be directed to the corresponding author/s.

## Ethics statement

The studies involving human participants were reviewed and approved by Seventh People's Hospital of Shanghai University of Traditional Chinese Medicine. The patients/participants provided their written informed consent to participate in this study.

## Author contributions

QW and YaW wrote the manuscript. YoW and QB completed data collection. FW and QZ designed and supervised the study. All authors contributed to the article and approved the submitted version.

## Funding

This study was supported by the grants from 2020 Health Science and Technology Project of Pudong New Area Health Commission (PW2020D-5), 2021 Scientific Research Project of Shanghai Municipal Commission of Health and Family Planning (No. 202140282), 2020 Science and Technology Development Fund of Pudong New Area Special Fund for People's Livelihood Scientific Research (PKJ2020-Y-15), and 2019 Scientific Research Project of Shanghai Science and Technology Commission (No. 19401972803).

## Conflict of interest

The authors declare that the research was conducted in the absence of any commercial or financial relationships that could be construed as a potential conflict of interest.

The handling editor declared a shared parent affiliation with the author QZ at the time of review.

## Publisher's note

All claims expressed in this article are solely those of the authors and do not necessarily represent those of their affiliated organizations, or those of the publisher, the editors and the reviewers. Any product that may be evaluated in this article, or claim that may be made by its manufacturer, is not guaranteed or endorsed by the publisher.

## References

[1] Ma H Campbell BC Parsons MW Churilov L Levi CR Hsu C. Thrombolysis guided by perfusion imaging up to 9 hours after onset of stroke. N Engl J Med. (2019) 380:1795–803. 10.1056/NEJMoa1813046 31067369

[2] Demel SL Stanton R Aziz YN Adeoye O Khatri P. Reflection on the past, present, and future of thrombolytic therapy for acute ischemic stroke. Neurology. (2021) 97(20 Suppl 2):S170-S7. 10.1212/WNL.0000000000012806 34785615

[3] Fonarow GC Smith EE Saver JL. Timeliness of tissue-type plasminogen activator therapy in acute ischemic stroke: patient characteristics, hospital factors, and outcomes associated with door-to-needle times within 60 min. Circulation. (2011) 123:750–8. 10.1161/CIRCULATIONAHA.110.974675 21311083

[4] Expert Committee of Stroke Prevention and Treatment Project of National Health Commission. Expert consensus on green Channel management of stroke during COVID-19 (2020).

[5] World Health Organization. WHO. Coronavirus (COVID-19) dashboard. (2022). Available online at: https://covid19whoint/ (accessed Jun 28, 2022).

[6] Wolter N Jassat W Walaza S Welch R Moultrie H Groome M. Early assessment of the clinical severity of the SARS-CoV-2 omicron variant in South Africa: a data linkage study. Lancet. (2022) 399:437–46. 10.1016/S0140-6736(22)00017-4 35065011 PMC8769664

[7] Tian D Sun Y Xu H Ye Q. The emergence and epidemic characteristics of the highly mutated SARS-CoV-2 Omicron variant. J Med Virol. (2022) 94:2376–83. 10.1002/jmv.27643 35118687 PMC9015498

[8] Morelli N Immovilli P Guidetti D. Letter by Morelli et al. regarding article, “Acute stroke care is at risk in the era of COVID-19: experience at a comprehensive stroke center in Barcelona”. Stroke. (2020) 51:e322–3. 10.1161/STROKEAHA.120.031124 33104482

[9] Hsiao J Sayles E Antzoulatos E Stanton RJ Sucharew H Broderick JP. Effect of COVID-19 on emergent stroke care: a regional experience. Stroke. (2020) 51:e2111–4. 10.1161/STROKEAHA.120.030499 32639860 PMC7359904

[10] Hoyer C Ebert A Huttner HB Puetz V Kallmünzer B Barlinn K. Acute stroke in times of the COVID-19 pandemic: a multicenter study. Stroke. (2020) 51:2224–7. 10.1161/STROKEAHA.120.030395 32516064

[11] Sui Y Luo J Dong C Zheng L Zhao W Zhang Y. Implementation of regional Acute stroke care map increases thrombolysis rates for acute ischaemic stroke in Chinese urban area in only 3 months. Stroke Vasc Neurol. (2021) 6:87–94. 10.1136/svn-2020-000332 32973114 PMC8005897

[12] Fassbender K Balucani C Walter S. Streamlining of prehospital stroke management: the golden h. Lancet Neurol. (2013) 12:585–96. 10.1016/S1474-4422(13)70100-5 23684084

[13] Siegler JE Zha AM Czap AL Ortega-Gutierrez S Farooqui M Liebeskind DS. Influence of the COVID-19 pandemic on treatment times for acute ischemic stroke: the society of vascular and interventional neurology multicenter collaboration. Stroke. (2021) 52:e104. 10.1161/STROKEAHA.120.032789 33250041 PMC7934334

[14] Candelaresi P Manzo V Servillo G Muto M Barone P Napoletano R. The impact of COVID-19 lockdown on stroke admissions and treatments in Campania. J Stroke Cerebrovasc Dis. (2021) 30:105448. 10.1016/j.jstrokecerebrovasdis.2020.105448 33166767 PMC7640890

[15] Powers WJ Rabinstein AA Ackerson T Adeoye OM Bambakidis NC Becker K American Heart Association Stroke Council. 2018 guidelines for the early management of patients with acute ischemic stroke: a guideline for healthcare professionals from the American Heart Association/American Stroke Association. Stroke. (2018) 49:e46-e110. 10.1161/STR.0000000000000158 29367334

